# SRPK1 Promotes Glioma Proliferation, Migration, and Invasion through Activation of Wnt/β-Catenin and JAK-2/STAT-3 Signaling Pathways

**DOI:** 10.3390/biomedicines12020378

**Published:** 2024-02-06

**Authors:** Mengna Shi, Dan Sun, Lu Deng, Jing Liu, Min-Jie Zhang

**Affiliations:** 1 Department of Oncology, Wenzhou Medical University, Wenzhou 325027, China; smn1501219274@163.com; 2 Department of Neurosurgery, The First Affiliated Hospital of Anhui University of Science and Technology (Huainan First People’s Hospital), Huainan 232002, China; 3 National Cancer Center/National Clinical Research Center for Cancer/Cancer Hospital, Chinese Academy of Medical Sciences and Peking Union Medical College, Beijing 100021, China; 18217721131@163.com; 4 State Key Laboratory of Molecular Oncology, National Cancer Center/National Clinical Research Center for Cancer/Cancer Hospital, Chinese Academy of Medical Sciences and Peking Union Medical College, Beijing 100021, China

**Keywords:** glioma, SRPK1, Wnt/β-catenin, JAK-2/STAT-3

## Abstract

Currently, the treatment of gliomas still relies primarily on surgery and radiochemotherapy. Although there are various drugs available, including temozolomide, the overall therapeutic effect is unsatisfactory, and the prognosis remains poor. Therefore, the in-depth study of the mechanism of glioma development and a search for new therapeutic targets are the keys to improving the therapeutic treatment of gliomas and improving the prognosis of patients. Immunohistochemistry is used to detect the expression of relevant molecules in tissues, qPCR and Western blot are used to detect the mRNA and protein expression of relevant molecules, CCK-8 (Cell Counting Kit-8) is used to assess cell viability and proliferation capacity, Transwell is used to evaluate cell migration and invasion ability, and RNA transcriptome sequencing is used to identify the most influential pathways. SRPK1 (SRSF protein kinase 1) is highly expressed in gliomas but is not expressed in normal tissues. Its expression is positively correlated with the grades of gliomas and negatively correlated with prognosis. SRPK1 significantly promotes the occurrence and development of gliomas. Knocking down SRPK1 leads to a significant decrease in the proliferation, migration, and invasion abilities of gliomas. Loss of SRPK1 expression induces G2/M phase arrest and mitotic catastrophe, leading to apoptosis in cells. Overexpression of SRPK1 activates the Wnt/β-catenin (wingless-int1/β-catenin) and JAK-2/STAT-3 (Janus kinase 2/signal transducer and activator of transcription 3) signaling pathways, promoting the proliferation, migration, and invasion of gliomas. Overexpression of SRPK1 rescues the reduced cell proliferation, migration, and invasion abilities caused by the silencing of β-catenin or JAK-2. A stable shRNA-LN229 cell line was constructed, and using a nude mouse model, it was found that stable knockout of SRPK1 significantly reduced the tumorigenic ability of glioma cells, as evidenced by a significant decrease in the subcutaneous tumor volume and weight in nude mice. We have demonstrated that SRPK1 is highly expressed in gliomas. Overexpression of SRPK1 activates the Wnt/β-catenin and JAK-2/STAT-3 signaling pathways, promoting the proliferation, migration, and invasion of gliomas. Silencing SRPK1-related signaling pathways may provide potential therapeutic options for glioma patients.

## 1. Introduction

Glioma is one of the most common malignant tumors in the nervous system [1,2], including low-grade gliomas (grades I–III) and high-grade gliomas (grade IV). The etiology of gliomas is not fully understood at present [3], but some studies suggest that genetic factors [4,5], environmental factors [6,7], and abnormal cell proliferation in the nervous system [8,9] may be related to their occurrence. The treatment of gliomas typically includes surgical resection of the tumor [10], radiotherapy, and chemotherapy as comprehensive treatment regimens. However, due to the different locations and malignancy levels of gliomas, treatment regimens may vary. Overall, the treatment outcomes are unsatisfactory, and the prognosis is poor. Therefore, there is still a need to continue to explore the molecular alterations that are associated with the malignant phenotype of gliomas and to find new and effective therapeutic targets.

SRPK1 is a serine/arginine protein kinase [11,12] that specifically acts on the SR (serine/arginine-rich) family of splicing factors. This protein is localized in the cell nucleus and plays an important role in regulating the splicing process of genes [13,14].

Studies have shown that SRPK1 plays an important role in promoting resistance to EGFR-TKI (epidermal growth factor receptor–tyrosine kinase inhibitor) [15,16] by enhancing the self-phosphorylation of GSK3β Ser9 (glycogen synthase kinase-3 beta) [17,18], which is independent of its kinase activity. Additionally, the expression of SRPK1 is correlated with the expression of EGFR in non-small-cell lung cancer patients, and the overexpression of SRPK1 is associated with EGFR (epidermal growth factor receptor) expression. SRPK1 affects the splicing process of genes by regulating the phosphorylation status of the splicing factors. This may lead to abnormal splicing of tumor-related genes, affecting the proliferation, migration, and drug resistance of tumor cells. The activity of SRPK1 is closely related to the expression and function of various tumor-related genes, including EGFR (epidermal growth factor receptor) [16], VEGF (vascular endothelial growth factor), and others [19,20]. These genes play important roles in the occurrence and development of tumors, and the abnormal activity of SRPK1 may affect the function of these genes. Research has found that SRPK1 is aberrantly expressed in both the tissue and cells of gliomas, while no abnormal expression has been observed in normal glioma cells [21]. Silencing SRPK1 has been shown to induce cisplatin resistance [21,22]. Under normoxic and moderately hypoxic conditions, knocking down SRPK1 inhibits the malignant progression of glioma cells [21,22], induces apoptosis in glioma cells [21,23], and suppresses AKT/E1F4E phosphorylation [21]. Plexin B1 promotes SRPK1 activity through the PI3K/AKT signaling pathway, thereby increasing cell growth, angiogenesis, and motility in vitro and in vivo [21,24]. However, the specific mechanism by which SRPK1 regulates glioma cell proliferation remains unclear, and there is still an urgent need to continue exploring SRPK1-related signaling pathways in gliomas to find new therapeutic approaches.

## 2. Methods

### 2.1. Materials

U118MG and LN229 cells were obtained from ATCC (USA), ruxolitinib from MCE: HY-50856, and mRFP-GFP-LC3 plasmid from HANBIO company, (Shanghai, China).

### 2.2. Cell Culture

U118MG and LN229 cells were cultured in DMEM (#8120359, Gibco) supplemented with 10% fetal bovine serum (#10100147, Gibco) and 1% penicillin-streptomycin (#15140148, Gibco) at 5% CO_2_ and 37 °C.

### 2.3. Neuroglial Tumor Tissue

A total of 210 glioma tissue samples were obtained from the medical oncology hospital, including 19 specimens taken from non-tumor tissues surrounding the glioma, or adjacent non-tumor tissues removed during the resection of deep tumor tissues and non-tumor tissues affected by edema surrounding high-grade gliomas. The grading of these gliomas was determined by two independent senior neurosurgical experts, and the diagnosis of all specimens was conducted according to the standards of the World Health Organization. Among them, the percentage of grade 2 gliomas was 12.6%, grade 3 gliomas was 15.0%, and grade 4 gliomas was 72.4%. All selected patients had no history of other malignant tumors, and all specimens were obtained from the remaining tissues after diagnosis with the informed consent of all patients. The specific clinical pathological information can be found in Table S2. This study was approved by the medical oncology hospital’s ethics committee (Ncc2014g12).

### 2.4. Study Design

Specific molecular markers with high expression in gliomas were screened using the TCGA database, followed by immunohistochemical analysis of SRPK1 expression in normal brain tissue, grade 3 gliomas, and grade 4 gliomas in human subjects to clarify the expression status of SRPK1. Subsequently, survival analysis and COX multivariate analysis were conducted on 210 glioma patients, and the results indicated that patients with high expression of SRPK1 had a poorer prognosis. The expression of SRPK1 was positively correlated with the grade of the gliomas. We selected normal human glioma cells U118 and LN229 and studied the effects of SRPK1 on glioma proliferation, invasion, and migration through knockdown and overexpression studies. Finally, RNA sequencing analysis demonstrated that SRPK1 can promote glioma proliferation, migration, and invasion by activating the Wnt/β-catenin and JAK-2/STAT-3 signaling pathways.

### 2.5. Study Setting

The aim of this study was to explore SRPK1 as a novel molecular marker that may become a new therapeutic target for gliomas. By setting up control and experimental groups, the study investigated the different effects of low and high expression of SRPK1 on glioma tissues and cells. The tissue samples were obtained from the hospital, and we obtained the corresponding ethical approval and informed consent from the patients. The quantity and characteristics of the samples met the research requirements, and convincing conclusions were obtained through COX multivariate analysis and related functional experiments.

### 2.6. Sample Size Determination

This study is exploratory research, and based on the previous experience of the research team, it is assumed that the correlation coefficient between key molecules in the SRPK1 pathway is 0.38, with a differential threshold of 0.1; a significance level of α = 0.05, β = 0.1; and a power of 0.90. Using PASS 11 software for calculation, a total of 162 tissue samples were required. Considering the possibility of unreadable results due to certain experimental conditions, the sample size was appropriately increased to 210 cases.

### 2.7. Immunofluorescence Microscopy

U118MG and LN229 cells were seeded on cell climbing plates and cultured until reaching a density of 60%. They were then fixed in 4% paraformaldehyde for 15 min. Subsequently, cell permeabilization was achieved using 10% Triton in PBS (phosphate-buffered saline) followed by blocking in 1% BSA (bovine serum albumin) for 1 h. The cells were incubated with a 1:100 dilution of CDH1 (E-cadherin), VIM (vimentin), α-tubulin (tubulin alpha-1A chain), and γ-tubulin (gamma tubulin) antibodies for 2 h. After washing with PBS, goat anti-rabbit IgG-Alex488 or goat anti-mouse IgG-Alex594 (1:100) (Abcam, CA, USA) were added and incubated in the dark on a shaker for 30 min. Following washing, DAPI (MCE) was added and incubated for 10 min. After washing, alcohol was added to evaporate the water, the climbing film was removed, 1 μL of fluorescence quencher was added, the cover was placed, and finally, images were captured using a fluorescence microscope.

### 2.8. Transfection of Cells with siRNA or Overexpression (OE) of Plasmid

The day before knockdown or overexpression, cells were seeded in a six-well plate to achieve a cell density of 40%. The culture medium was then removed, and the cells were washed twice with sterile PBS. Each well of the six-well plate was filled with 1.5 mL of Opti-MEMTM medium (Gibco, USA). Additionally, a sterile centrifuge tube was prepared, and 0.5 mL of Opti-MEMTM medium, 5μL of small interfering RNA (siRNA) or overexpression plasmid, and 5μL of Lipofectamine 2000 (Thermo Fisher Scientific, Shanghai, China) were mixed and allowed to stand for 20 min. The mixture was then added to the experimental group cells, while the control group received the same mixture without siRNA or overexpression plasmid. After 6 h, the medium was replaced with complete culture medium, and the cells were cultured for 48 h before collection for analysis.

### 2.9. Immunohistochemistry

The fresh paraffin-embedded tissue sections were placed in a 65 °C oven for 15 min for deparaffinization and baking. Subsequently, the tissue sections were transferred to xylene for 30 min of deparaffinization, followed by dehydration in 85%, 95%, and 100% anhydrous ethanol. The deparaffinized sections were then subjected to high-pressure citrate repair, followed by immersion in cold water and addition of a small amount of H_2_O_2_ in the dark for 10 min. After thorough washing, the sections were subjected to DAB (3,3′-Diaminobenzidine) staining for 3 min, followed by hematoxylin staining. The hematoxylin-stained sections were placed in water for 5 min, followed by gradient dehydration in anhydrous ethanol for 5 min. The dehydrated tissue sections were air-dried, and finally, slide sealing and observation were performed.

### 2.10. RNA Extraction and qPCR

The following procedure was used: Collect U118MG and LN229 cells, lyse with Trizol (Invitrogen, USA), centrifuge to collect cell suspension, and incubate at room temperature for 5 min. Add 1 mL of Trizol to 0.2 mL chloroform, shake for 10 s, and incubate at room temperature for 3 min. Centrifuge at 4 °C for 15 min, transfer the upper clear liquid to a new tube, wash RNA precipitation with 75% ethanol, centrifuge at 5000× *g* at 4 °C for 5 min, discard the supernatant, air dry or vacuum dry the RNA precipitation at room temperature for approximately 5–10 min. Measure RNA concentration. Primer sequences are shown in Supplementary Table S3 (GenePharma, Suzhou, China).

### 2.11. Cell Proliferation Assay

U118MG and LN229 cells were cultured in MEM (Gibco) medium containing 10% fetal bovine serum (FBS) and 1% double antibodies (penicillin/streptomycin), respectively, and placed in an incubator at 37 °C with 5% CO_2_. Both cells were laid flat on a six-well plate, and densities of 40% were treated with siRNA-SRPK1 administration for 24 h and for crystalline violet (Beijing Solarbio Science and Technology, Beijing, China) staining. In addition, both cells were spread flat in 96-well plates according to 2 × 10^3^ cells per well, treated by administration at the same concentrations as above, and assayed for cell activity every 24 h using CCK-8 (MCE).

### 2.12. RNA-Seq

RNA-seq was performed by Aksomics Inc. (Shanghai, China). In brief, RNA-seq was performed on the Illumina NovaSeq 6000 platform. FPKM values of gene and transcript levels were calculated. Hierarchical clustering, gene ontology, path analysis, scatter plots, and volcano plots were performed using differentially expressed genes in Python (v3.11.3), or Shell environments for statistical calculation and graphical display.

### 2.13. Flow Cytometry

The following procedure was used: Collect the suspension of transfected U118MG and LN229 cells and perform cell counting. Dispense 1 × 10^6^ cells into each 1.5 mL EP tube and centrifuge at 3500 rpm for 2 min at 4 °C. Then, resuspend in 100 µL PBS, add 10 µL serum for blocking, and incubate at 4 °C in the dark for 30 min. Add the corresponding fluorescently labeled antibody, mix the solution, and incubate at 4 °C in the dark for 30 min. Wash twice with PBS, resuspend in 200 µL PBS, filter through gauze into a flow tube, and analyze the samples using a flow cytometer (Bio-Rad, Inc, Hercules, CA, USA).

### 2.14. Western Blot

Total proteins were separated using RIPA buffer (#P0013B, Beyotime, Shanghai, China) in the presence of protease inhibitor mixture. The protein concentration of the lysate was determined using BCA protein assay kit (#P0009, Beyotime, Shanghai, China), separated with polyacrylamide gel electrophoresis, and transferred to PVDF membranes. The lysates were then blocked with 5% skim milk for 1 h and incubated with primary antibody at 4 °C overnight. After washing with TBST, the secondary antibodies were incubated at room temperature for 2 h. For antibody information, see Supplementary Table S1.

### 2.15. Transwell Infiltration Experiment

The invasion assay was performed using Boyden chambers filtered with Transwell membranes (#3422, Corning Costar). A certain amount of U118MG and LN229 cells were inoculated into the chambers for the invasion assay, and the incubation period was 48 h. Cells that did not penetrate the filter were wiped off, and cells on the lower surface of the filter were stained with 4% crystalline violet. Finally, the number of invading cells was counted under a light microscope.

### 2.16. Monomer Red Fluorescent Protein (mRFP)—Green Fluorescent Protein (GFP)—LC3 Tandem Fluorescence Protein Quenching Test

U118MG and LN229 cells were inoculated onto cell-climbing plates, and after the cell density reached 60%, the cells were transfected with mRFP-GFP-LC3 packaging plasmid and Lipofectamine 3000 using amounts of 1 µg and 3 µL, respectively. After continuing to incubate for 6 h, the experimental group was switched to dosing medium while the control group continued to be incubated with the complete medium. After 48 h of incubation, the cells were fluorescently photographed with a confocal microscope LSM 700 (Carl Zeiss) and analyzed with ZEN version 3.0 software.

### 2.17. In Vivo Experiments

Animal experiments were performed according to the protocol approved by the Animal Ethics Committee of the Second Affiliated Hospital of Wenzhou Medical University (WYDW 2022-0700). For the subcutaneous xenograft model, twelve female BALB/c nude mice of 4–6 weeks of age (Beijing Dynamic River Laboratory Animal Technology Co., Ltd.) were randomly divided into two groups. The normal group was injected subcutaneously with LN229 cells, and the experimental group was injected with LN229 cells stably knocked down from SRPK1 (SRPK1-shRNA, each injected with 2 × 10^6^ cells). Tumor size was measured every 5 days, and the mice were euthanized at the end of the 25-day experiment. The tumors were then dissected, weighed, and analyzed. Tumor size was calculated using the formula: (width)^2^ × length × 0.52. For the orthotopic model, nude mice (weekly age: 4–6 weeks) were randomly divided into two groups of three mice each and labeled as control and experimental groups. Firstly, the experimental nude mice were continuously anesthetized using isoflurane. Then, 5 μL of LN229 cell suspension (total number of cells: 5 × 10^5^) was injected into the caudate nucleus of the brain of each nude mouse in the control group, and 5 μL of SRPK1-shRNA-LN229 cell suspension (total number of cells: 5 × 10^5^) was injected into the caudate nucleus of the brain of each nude mouse in the experimental group. After four weeks of culture, the T2 weight (T2W) of the xenografts in the nude mice’s cranium was detected using a 7.0 T MRI scanner (Bruker BioSpin, Billerica, MA, USA).

### 2.18. Statistical Analysis

The results of this study are expressed as the mean ± SD of at least three independent experiments and were analyzed using GraphPad Prism 8 (GraphPad Software Inc., San Diego, CA, USA). Two-factor ANOVA tests were used to examine significant differences between any two groups, whereas one-way ANOVA was followed by post hoc Tukey’s multiple comparison for statistical significance between three or more groups. *p* < 0.05 was considered statistically significant.

## 3. Results

### 3.1. SRPK1 Overexpression Was Positively Correlated with Glioma Grade and Negatively Correlated with Patient Prognosis

**Figure 1:**
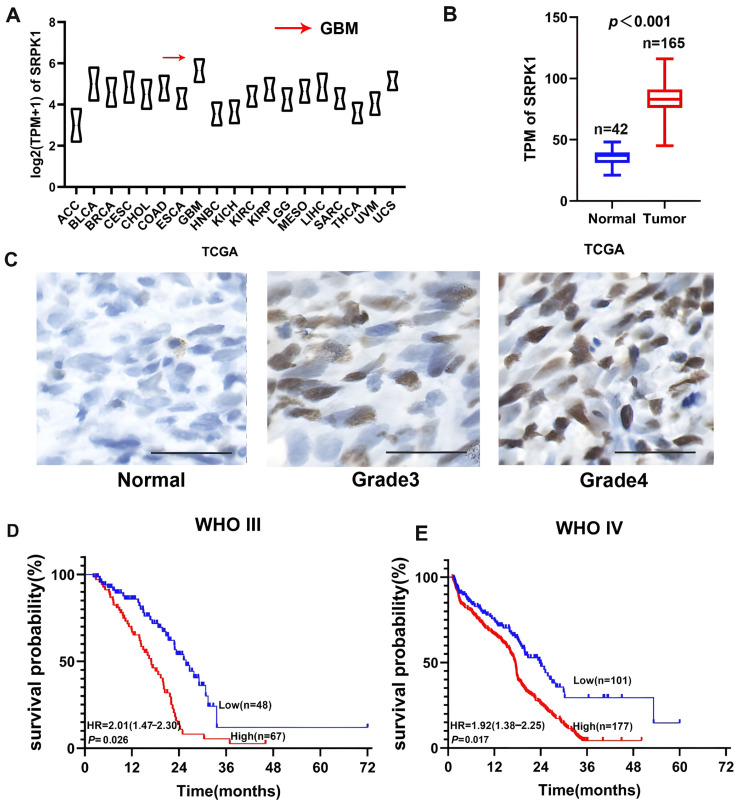
SRPK1 overexpression was positively correlated with glioma grade and negatively correlated with patient prognosis. Analysis of SRPK1 mRNA expression in various cancer samples from the TCGA database (**A**). Analysis of SRPK1 mRNA expression in glioma samples and normal tissues from the TCGA database (**B**). Expression of SRPK1 in normal brain tissue and glioma specimens, with Figure 1, Figure 2 and Figure 3 showing normal brain tissue, grade 3 glioma, and grade 4 glioma. Scale bar = 100 μm (200×, (**C**)). Patients with high expression of SRPK1 in WHO grade III gliomas had a worse prognosis than those with low expression of SRPK1 (*p* = 0.026, (**D**)). Patients with high expression of SRPK1 in WHO grade IV gliomas had a worse prognosis than those with low expression of SRPK1 (*p* = 0.017, (**E**)).

Analysis of the Cancer Genome Atlas (TCGA) database revealed that SRPK1 expression is highest in glioblastoma among 19 types of cancer (Figure 1A). Compared with normal tissues (*n* = 37), TCGA glioblastoma (*n* = 192) showed a significant increase in SRPK1 mRNA (Figure 1B). Subsequently, we conducted immunohistochemical experiments on normal brain tissue and various grades of gliomas. Immunohistochemical analysis indicates that SRPK1 is expressed at low levels or not expressed in normal brain tissue, while it is highly expressed in grade IV gliomas and is expressed to a lesser extent in grade III gliomas (Figure 1C), with expression levels positively correlated with glioma grading (Figure 1D). Notably, SRPK1 expression was most significant in WHO grade IV gliomas, while it was not expressed in normal brain tissue (Figure 1C). High-intensity immunohistochemistry results demonstrated that SRPK1 is positively associated with poor prognosis in grade III gliomas (HR = 2.01, 95% CI 1.47–2.30, *p* = 0.026) and grade IV gliomas (HR = 1.92, 95% CI 1.38–2.25, *p* = 0.017) (Figure 1E). A multifactorial COX analysis of WHO grade IV gliomas combined with the patients’ preoperative KPS scores and postoperative radiotherapy showed that, similar to postoperative adjuvant radiotherapy, high expression of DYRK2 was an independent prognostic risk factor for WHO grade IV gliomas (HR = 1.55, 95% CI: 1.05–2.54, *p* = 0.003), and there was no correlation with the patients’ gender, age, etc. (*p* > 0.05) (Table 1).

### 3.2. Low Expression of SRPK1 Inhibits Glioma Cell Viability and Proliferation

**Figure 2:**
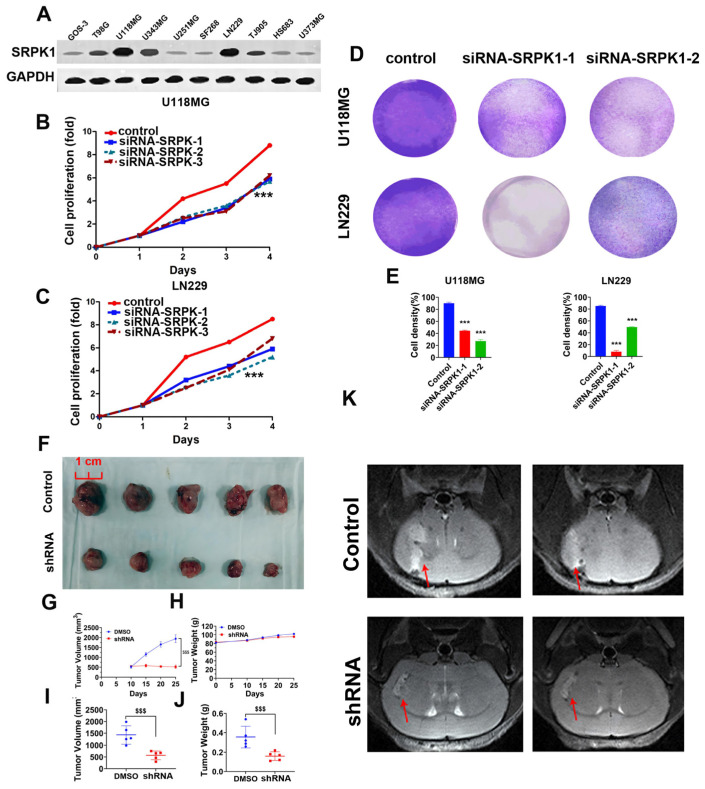
Low expression of SRPK1 inhibits glioma cell viability and proliferation. Western blot analysis of SRPK1 expression in ten glioma cell lines (**A**). CCK-8 assay to detect the proliferation ability of U118MG cells after knocking out SRPK1 (**B**). CCK-8 assay to detect the proliferation ability of LN229 cells after knocking out SRPK1 (**C**). Colony formation experiment after knocking out SRPK1 in U118MG and LN229 cell lines (**D**) and quantitative results (**E**), mean ± SD, *n* = 3, *** *p* < 0.001 for the knockout group vs. control. Subcutaneous xenograft tumor formation experiment in nude mice (shRNA: shRNA-SRPK1, (**F**)). Changes in subcutaneous tumor volume over time (**G**), ^$$$^ *p* < 0.001 for the shRNA group vs. control. Changes in body weight of nude mice over time (**H**). Measurement of subcutaneous tumor volume (**I**), ^$$$^ *p* < 0.001 for the shRNA group vs. control. Measurement of subcutaneous tumor weight (**J**), ^$$$^ *p* < 0.001 for the shRNA group vs. control. T2-weighted MRI analysis representative images (shRNA: shRNA-SRPK1, (**K**)).

To clarify the expression of SRPK1 in various glioma cell lines, we selected 10 glioma cell lines and performed immunoblotting. The results indicated significant expression of SRPK1 in U118MG, LN229, T98G, U343MG, and TJ905 cells (Figure 2A). We chose to proceed with further studies using U118MG and LN229 cells. Subsequently, we knocked out SRPK1 in U118MG and LN229 cells and detected changes in cell proliferation using CCK-8. The results showed that knocking out SRPK1 significantly inhibited the proliferation of U118MG and LN229 cells (Figure 2B,C). The results of the colony formation experiment also demonstrated a significant reduction in the clonogenic capacity of the U118MG and LN229 cells after SRPK1 knockdown (Figure 2D,E). Furthermore, we constructed SRPK1-shRNA LN229 cells and conducted subcutaneous tumorigenesis experiments in nude mice. The results indicated a significant decrease in tumorigenic ability after SRPK1 knockout compared with the control group, as evidenced by a reduction in subcutaneous tumor volume and weight (Figure 2F–J). T2-weighted MRI (magnetic resonance imaging) analysis revealed a significantly smaller intracranial tumor volume in SRPK1-shRNA cells compared with normal tumor cells (Figure 2K).

### 3.3. Silencing SRPK1 will Inhibit Glioma Cell Migration and Invasion

**Figure 3:**
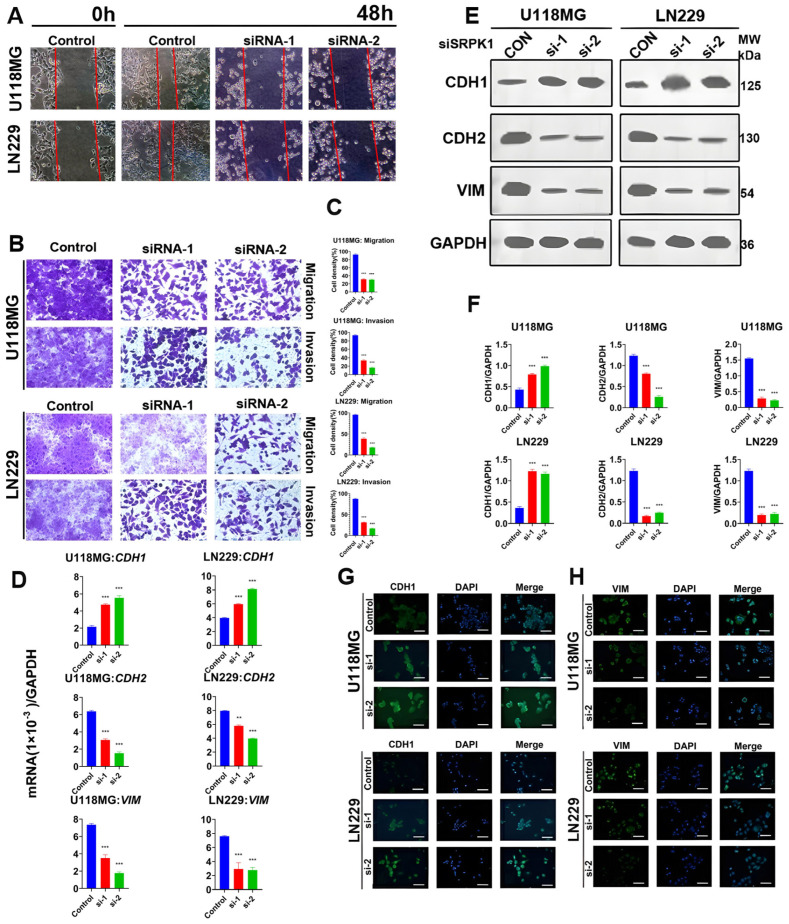
Silencing SRPK1 will inhibit glioma cell migration and invasion. Results of the scratch assay in U118MG and LN229 cell lines after knocking out SRPK1 (48 h, (**A**)). Transwell assay results in U118MG and LN229 cell lines after knocking out SRPK1 (48 h, (**B**)) and quantitative results (**C**). qPCR detection of the expression of invasion and migration key molecules in U118MG and LN229 cells after knocking out SRPK1 (**D**), ** *p* < 0.01, *** *p* < 0.001 for the knockout group vs. control. Western blot detection of the expression of invasion and migration key molecules in U118MG and LN229 cells after knocking out SRPK1 (**E**), ** *p* < 0.01, *** *p* < 0.001 for the knockout group vs. control, and quantitative results (**F**). Immunofluorescence detection of the expression of invasion and migration key molecules in U118MG and LN229 cells after knocking out SRPK1. Scale bar = 50 μm (**G**,**H**).

The results of the cell scratch assay showed that 48 h after knocking out SRPK1 in U118MG and LN229 cells, the cells at the edge of the scratch were relatively distant from the center of the scratch (Figure 3A), indicating a lower ability to recover the scratch. Furthermore, the Transwell assay results demonstrated that after knocking out SRPK1, the transmembrane ability of the U118MG and LN229 cells significantly decreased, with a notable reduction in the number of cells translocating across the membrane (Figure 3B,C). The CDH1 gene is involved in regulating cell adhesion, migration, and epithelial cell proliferation. Its functional loss leads to increased cell invasion and migration. CDH2 (N-cadherin), also known as neural cadherin (NCAD), is a protein encoded by the CDH2 gene. Increased CDH2 expression signifies enhanced invasion and migration ability of tumor cells. The vimentin gene encodes a type III intermediate filament protein, which, along with microtubules and actin microfilaments, forms the cell’s cytoskeletal structure and is involved in cell migration, invasion, and metastasis. The decrease in the invasion and migration ability of tumor cells is often accompanied by increased expression of the core molecule CDH1, decreased expression of CDH2, and decreased expression of VIM. The results of qPCR (Figure 3D) and Western blot (Figure 3E,F) showed that after knocking out SRPK1, the mRNA and protein expression of the migration and invasion key molecule CDH1 were upregulated in the U118MG and LN229 cells, while the mRNA and protein expression of CDH2 and VIM were downregulated. Immunofluorescence results were similar, showing increased expression of CDH1 and decreased expression of VIM after knockdown of SRPK1 (Figure 3G,H). The cells were stained for the microfilament-associated protein SYNPO (synaptopodin) and the focal adhesion-related protein PXN (paxillin). The results indicated that the low expression of SYNPO and PXN predicted a decrease in cell invasion and migration ability. After knocking out SRPK1, the expression of SYNPO and PXN significantly decreased (Figure S1), suggesting that inhibiting the expression of SRPK1 reduced the migration and invasion ability of the U118MG and LN229 cells.

### 3.4. Lack of SRPK1 Expression Induces Apoptosis and G2/M Phase Blockade

**Figure 4:**
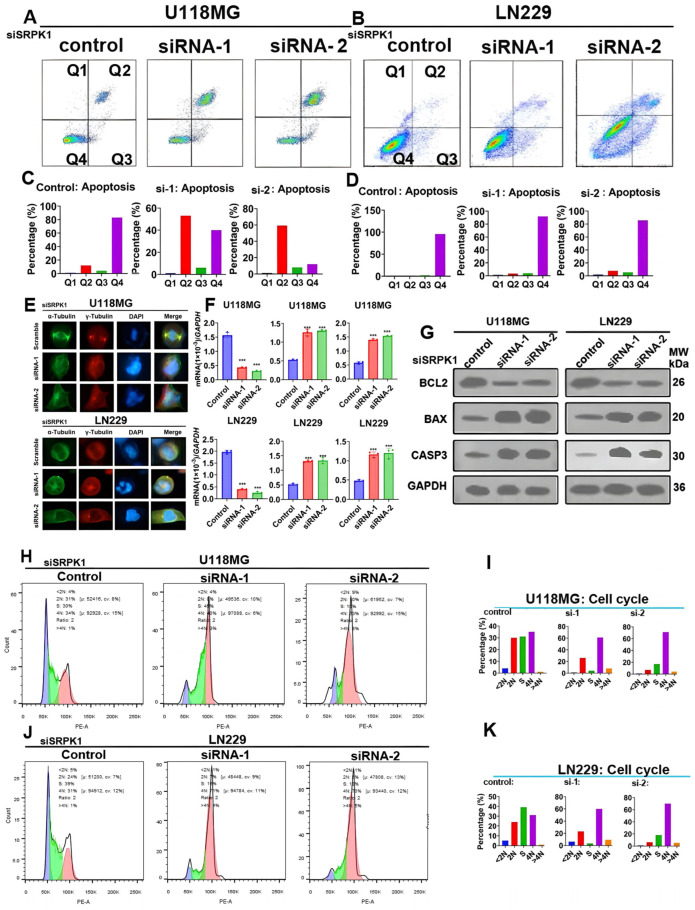
Lack of SRPK1 expression induces apoptosis and G2/M phase blockade. Flow cytometry detection of apoptosis in U118MG and LN229 cells, after knocking out SRPK1 (**A**,**B**), and quantitative results (**C**,**D**). Immunofluorescence staining of α-tubulin and β-tubulin in U118MG and LN229 cells after knocking out SRPK1. Scale bar = 50 μm. (**E**). qPCR detection of the mRNA levels of apoptosis-related molecules in U118MG and LN229 cell lines after knocking out SRPK1 (**F**), *** *p* < 0.001 for the knockout group vs. control. qPCR detection of the protein levels of apoptosis-related molecules in U118MG and LN229 cell lines after knocking out SRPK1 (**G**). G2/M phase arrest in U118MG and LN229 cells after knocking out SRPK1 (**H**,**J**). The quantitative results (**I**,**K**).

The flow cytometry analysis indicated a significant increase in the number of apoptotic cells in the U118MG and LN229 cells after knocking out SRPK1 (Figure 4A–D). Immunofluorescence results for α-tubulin and γ-tubulin suggested that the loss of SRPK1 expression caused disorganization of the centrosome in the U118MG and LN229 cells, leading to multipolar spindle formation and spindle rupture, resulting in mitotic catastrophe (Figure 4E) and subsequent cell apoptosis. Additionally, the mRNA and protein expression of the apoptosis-related molecules Bax (Bcl-2-associated X protein) and caspase3 (cysteine-aspartic proteases 3) were upregulated (Figure 4F), while the mRNA and protein expression of BCL2 were downregulated (Figure 4G). Cell cycle analysis results also indicated that the reduced expression of SRPK1 induced mitotic arrest at the G2/M phase (Figure 4H–K). Electron microscopy observation after knocking out SRPK1 revealed the presence of multiple apoptotic vesicles in the cells (Figure S2), leading to mitotic arrest and cell apoptosis.

### 3.5. SRPK1 Promotes Glioma Proliferation and Migration through Activation of Wnt/β-Catenin and JAK-2/STAT-3 Signaling Pathways, Invasion

**Figure 5:**
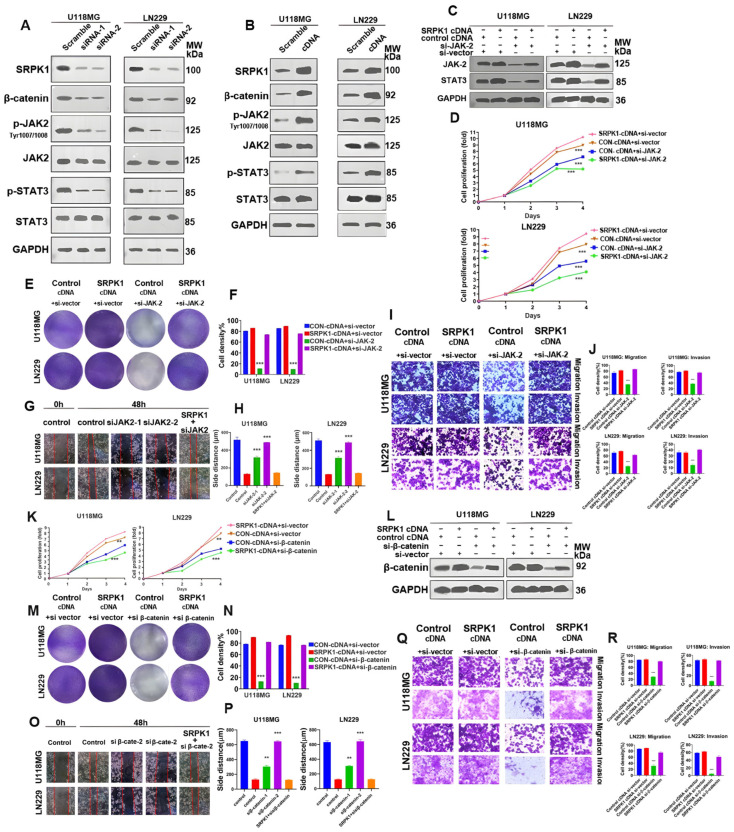
SRPK1 promotes glioma proliferation and migration through the activation of Wnt/β-catenin and JAK-2/STAT-3 signaling pathways. Western blot detection of the protein levels of key molecules in the Wnt/β-catenin and JAK-2/STAT-3 signaling pathways in U118MG and LN229 cells Knockdown SRPK1 (**A**). Western blot detection of the protein levels of key molecules in the Wnt/β-catenin and JAK-2/STAT-3 signaling pathways in U118MG and LN229 cells overexpressing SRPK1 (**B**). Western blot detection of the protein levels of key molecules in the JAK-2/STAT-3 signaling pathway in U118MG and LN229 cells overexpressing SRPK1 (SRPK1 cDNA), knocking out JAK-2 (si-JAK-2), or overexpressing SRPK1 followed by knocking out JAK-2 (**C**). CCK-8 assay to detect the proliferation ability of U118MG and LN229 cells overexpressing SRPK1, knocking out JAK-2, or overexpressing SRPK1 followed by knocking out JAK-2 (SRPK1 cDNA+si-vector: overexpression of SRPK1+ control vector, CON-cDNA+si-vector: normal group with overexpression and knockout vector, CON-cDNA+siJAK-2: normal group with overexpression vector and JAK-2 knockout, SRPK1 cDNA+siJAK-2: overexpression of SRPK1 followed by JAK-2 knockout) (**D**). Colony formation ability of U118MG and LN229 cells overexpressing SRPK1 or knocking out JAK-2 or overexpressing SRPK1 followed by knocking out JAK-2 (**E**) and quantitative results (**F**), *** *p* < 0.001 for the experimental group vs. control. Scratch assay results in U118MG and LN229 cells overexpressing SRPK1 or knocking out JAK-2 or overexpressing SRPK1 followed by knocking out JAK-2 (**G**). The quantitative results (**H**). Transwell assay results in U118MG and LN229 cells overexpressing SRPK1, knocking out JAK-2, or overexpressing SRPK1 followed by knocking out JAK-2, representative images (**I**) and quantitative results (**J**), *** *p* < 0.001 for JAK-2 knockout group vs. control. CCK-8 assay to detect the proliferation ability of U118MG and LN229 cells overexpressing SRPK1, knocking out β-catenin, or overexpressing SRPK1 followed by knocking out β-catenin (**K**). Western blot detection of the protein levels of key molecules in the Wnt/β-catenin signaling pathway in U118MG and LN229 cells overexpressing SRPK1 (SRPK1 cDNA), knocking out β-catenin (si-β-catenin), or overexpressing SRPK1 followed by knocking out β-catenin (**L**). Colony formation ability of U118MG and LN229 cells overexpressing SRPK1, knocking out β-catenin, or overexpressing SRPK1 followed by knocking out β-catenin (**M**) and quantitative results (**N**), ** *p* < 0.01, *** *p* < 0.001 for the experimental group vs. control. Scratch assay results in U118MG and LN229 cells overexpressing SRPK1, knocking out β-catenin, or overexpressing SRPK1 followed by knocking out β-catenin (**O**) and quantitative results (**P**). Transwell assay results in U118MG and LN229 cells overexpressing SRPK1, knocking out β-catenin, or overexpressing SRPK1 followed by knocking out β-catenin: representative images (**Q**) and quantitative results (**R**), *** *p* < 0.001 for β-catenin knockout group vs. control.

To elucidate how SRPK1 affects the occurrence of glioma, we conducted RNA transcriptome sequencing of the normal cell group and the SRPK1-shRNA-LN229 cell group. The results indicated significant enrichment of upregulated and downregulated proteins in the Wnt/β-catenin pathway (Figure S3) followed by the JAK-2/STAT-3 signaling pathway (Figure S4). Western blot results preliminarily confirmed the regulatory effect of SRPK1 expression changes on the Wnt/β-catenin and JAK-2/STAT-3 pathways. Knocking out SRPK1 reduced the levels of Wnt3a, β-catenin, p-JAK-2 (Tyr1007/1008), and p-STAT3 in U118MG and LN229 cells (Figure 5A), while the overexpression of SRPK1 upregulated the levels of Wnt3a, β-catenin, p-JAK-2 (Tyr1007/1008), and p-STAT3 (Figure 5B).

To clarify how SRPK1 regulates the JAK-2/STAT-3 signaling pathway, JAK-2 silencing significantly reduced the activity of the JAK-2/STAT-3 pathway, and SRPK1 overexpression partially restored the decrease in p-JAK-2 (Tyr1007/1008) and p-STAT3 in the si-JAK-2-U118MG and si-JAK-2-LN229 cells (Figure 5C). Growth curve (Figure 5D) and colony formation experiments (Figure 5E,F) indicated that SRPK1 overexpression reversed the decreased clonal proliferation ability of the si-JAK-2-U118MG and si-JAK-2-LN229 cells. A cell scratch assay (Figure 5G,H) and Transwell assay also demonstrated that SRPK1 overexpression partially rescued the weakened cell invasion and migration ability caused by knocking out JAK-2 (Figure 5I,J).

**Figure 6:**
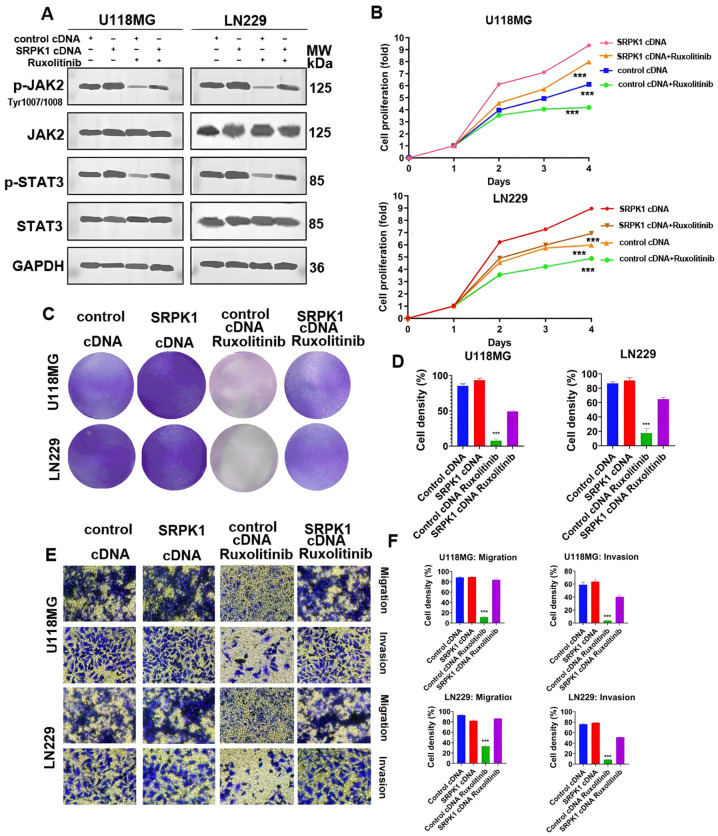
Western blot detection of the protein levels of key molecules in the JAK-2/STAT-3 signaling pathway in U118MG and LN229 cells overexpressing SRPK1, treated with ruxolitinib, or overexpressing SRPK1 followed by ruxolitinib treatment (**A**). CCK-8 assay to detect the proliferation ability of U118MG and LN229 cells overexpressing SRPK1, treated with ruxolitinib, or overexpressing SRPK1 followed by ruxolitinib treatment (**B**), *** *p* < 0.001. Colony formation assay results in U118MG and LN229 cells overexpressing SRPK1, treated with ruxolitinib, or overexpressing SRPK1 followed by ruxolitinib treatment: representative images (**C**). The quantitative results (**D**), *** *p* < 0.001 for ruxolitinib treatment group vs. control. Transwell assay results in U118MG and LN229 cells overexpressing SRPK1, treated with ruxolitinib, or overexpressing SRPK1 followed by ruxolitinib treatment: representative images (**E**). The quantitative results (**F**), *** *p* < 0.001 for ruxolitinib treatment group vs. control.

Furthermore, treatment with the JAK-2 inhibitor ruxolitinib at 100 nM for 48 h significantly reduced the activity of the JAK-2/STAT-3 signaling pathway (Figure 6A). After ruxolitinib treatment, the growth curve (Figure 6B) and colony formation ability (Figure 6C) of the U118MG and LN229 cells were consistent with the results of JAK-2 knockout. SRPK1 overexpression rescued the decreased cell proliferation (Figure 6B–D), Migration and invasion ability (Figure 6E,F) caused by ruxolitinib treatment.

In addition, we investigated the impact of silencing β-catenin on the progression of glioma cells. CCK-8 assay results showed that SRPK1 overexpression significantly enhanced cell proliferation, while knocking out β-catenin significantly inhibited cell proliferation. SRPK1 overexpression rescued the decreased cell proliferation caused by knocking out β-catenin (Figure 5K). SRPK1 overexpression upregulated the protein expression of β-catenin, even in cells where β-catenin was knocked out, enhancing the protein expression level of β-catenin (Figure 5L). Colony formation experiments (Figure 5M,N), cell scratch assay (Figure 5O,P), and Transwell analysis (Figure 5Q,R) indicated that SRPK1 overexpression reversed the decreased clonal proliferation, migration, and invasion ability of si-β-catenin-U118MG and si-β-catenin-LN229 cells.

In summary, these results indicate that the Wnt/β-catenin and JAK-2/STAT-3 signaling pathways are regulated by SRPK1. SRPK1 promotes the proliferation, migration, and invasion of gliomas through the activation of the Wnt/β-catenin and JAK-2/STAT-3 signaling pathways.

### 3.6. The Expression Levels of SRPK1 and Related Molecules in Subcutaneous Graft and Glioma Tissues of Nude Mice Were Positively Correlated

**Figure 7:**
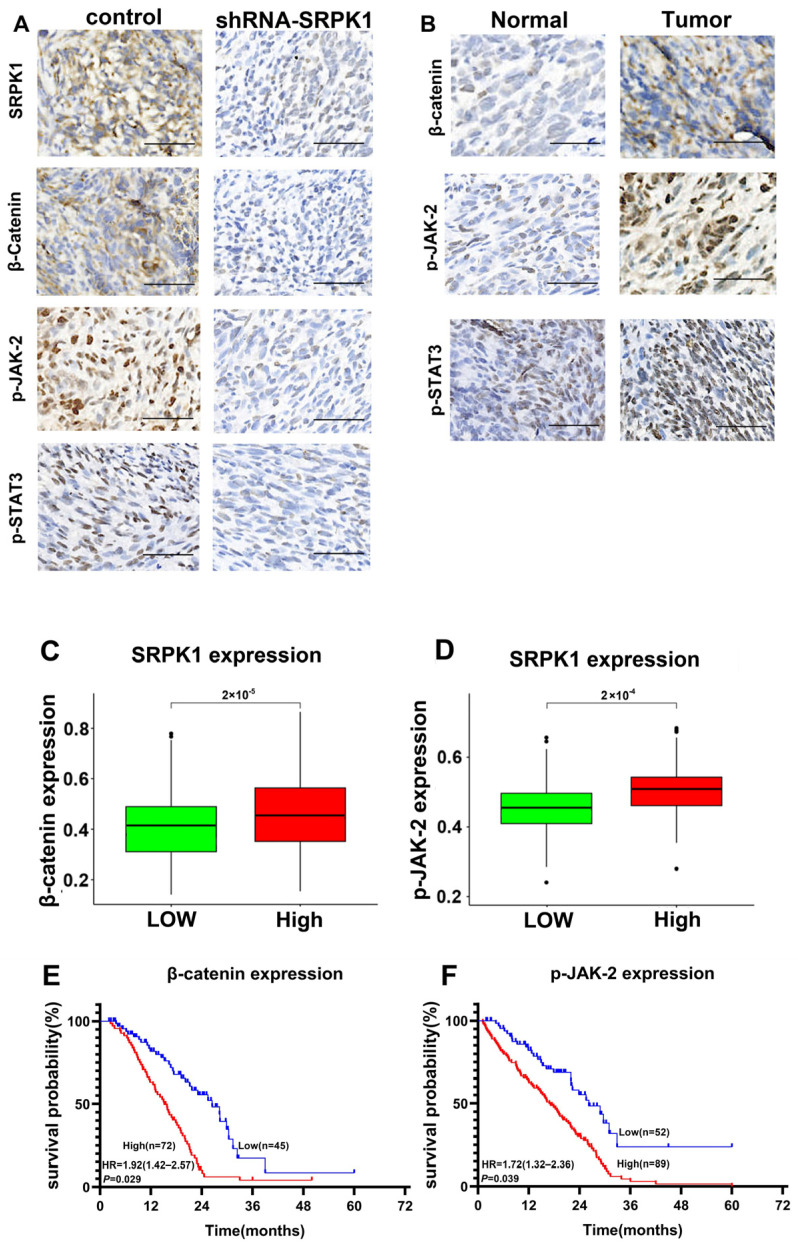

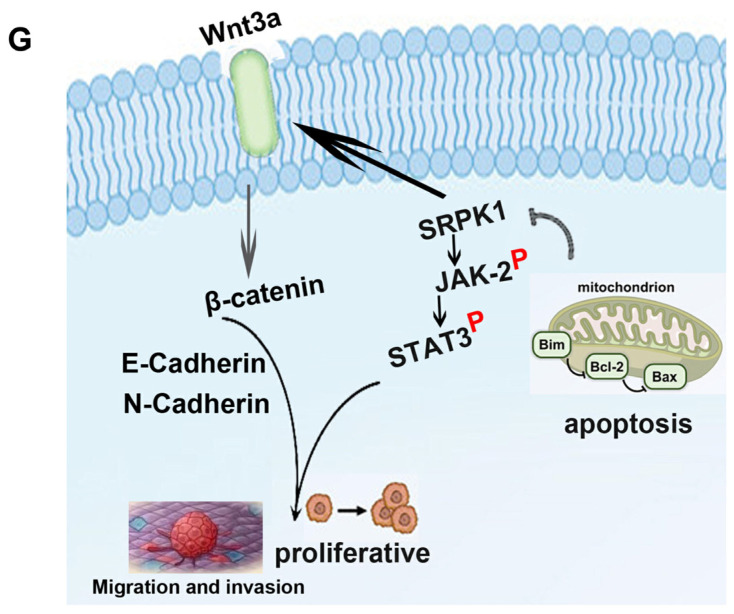
The expression levels of SRPK1 and related molecules in subcutaneous graft and glioma tissues of nude mice were positively correlated. Immunohistochemistry to detect the expression levels of key molecules in the Wnt/β-catenin and JAK-2/STAT-3 signaling pathways in subcutaneous xenograft tumors in nude mice. Scale bar = 100 μm (**A**). Expression levels of key molecules in the Wnt/β-catenin and JAK-2/STAT-3 signaling pathways in normal human brain tissues and gliomas (**B**). Expression of β-catenin and p-STAT3 in gliomas with high and low expression of SRPK1 (**C**,**D**). Survival analysis of β-catenin and p-STAT3 overexpression in grade IV glioblastoma. Schematic diagram illustrating the promotion of glioma progression by SRPK1 through the Wnt/β-catenin and JAK-2/STAT-3 signaling pathways (**E**,**F**). SRPK1 Promotes Glioma Proliferation, Migration, and Invasion through Activation of Wnt/β-Catenin and JAK-2/STAT-3 signaling pathways (**G**).

Immunohistochemical experiments detected the expression of SRPK1 downstream, β-catenin, p-JAK-2, and p-STAT3 in subcutaneous tumor tissues of nude mice. The results indicated that stable knockdown of SRPK1 significantly reduced the expression of β-catenin, p-JAK-2, and p-STAT3 (Figure 7A). The expression of β-catenin, p-JAK-2, and p-STAT3 in normal human brain tissue was much lower than that in glioma tissues (Figure 7B). Furthermore, high expression of SRPK1 in glioma tissues often correlated with high expression of β-catenin and p-JAK2 (Figure 7C,D). Survival curve analysis showed a negative correlation between the expression of β-catenin and p-JAK-2 and the survival time of patients (Figure 7E,F). Based on these findings, we speculate that the activation of the Wnt/β-catenin and JAK-2/STAT-3 signaling pathways by SRPK1 promotes the proliferation, migration, and invasion of gliomas (Figure 7G).

## 4. Discussion

The study of molecular markers in gliomas is currently a hot topic in the field of tumor research. By studying molecular markers in glioma tissues, we can better understand the mechanisms of tumor development, prognosis assessment, and the formulation of personalized treatment plans. Current research has identified therapeutic methods such as TERT (telomerase reverse transcriptase) promoter mutations [23,24,25], IDH1/2 (isocitrate dehydrogenase 1) mutations, and 1p/19q co-deletion [26,27]; corresponding treatments have been applied in clinical practice. Despite achieving a series of treatment outcomes, the prognosis remains poor. In this study, we have demonstrated that SRPK1 is a potential therapeutic target for gliomas. We observed high expression of SRPK1 in glioma tissues, while it is not expressed in normal tissues. The expression of SRPK1 is positively correlated with the grade of glioma and negatively correlated with patient prognosis, serving as an independent indicator for glioma prognosis assessment.

The protein encoded by the SRPK1 gene is a serine/arginine-rich protein kinase, which plays an important role in regulating the pre-mRNA splicing process. Specifically, the SRPK1 protein kinase is involved in the phosphorylation modification of splicing factors, thereby affecting the activity of splicing factors and the process of alternative splicing. This is crucial for the regulation of gene expression and the normal functioning of cellular processes. Additionally, SRPK1 has been found to play an important role in the growth, metastasis, and transformation of tumors [28,29]. It is closely associated with the occurrence of various tumors, including but not limited to breast cancer [30,31], colorectal cancer [32], lung cancer [33], and cervical cancer [34]. Therefore, the function of the SRPK1 gene is not only important in normal cellular physiological processes but also significant in the occurrence and development of tumors. In this study, we found that SRPK1 plays an important role in the development of gliomas. Knocking down SRPK1 significantly reduced the proliferation, migration, and invasion abilities of the glioma cell lines U118MG and LN229. Furthermore, silencing SRPK1 significantly induced G2/M phase arrest and mitotic catastrophe, leading to apoptosis in the U118MG and LN229 cell lines. Similar results were obtained in other glioma cell lines, such as a significant decrease in proliferation ability in U343 cells after silencing SRPK1 (Figure S5) and a significant inhibition of migration and invasion abilities (Figures S6 and S7). These results all indicate that SRPK1 plays an important role in the occurrence and development of gliomas.

Studies have reported that SRPK1 is aberrantly expressed in gliomas, while no significant abnormal expression is observed in normal glioma cells [21,22]. The high expression of SRPK1 promotes the malignant phenotype of glioma cells [22], while silencing SRPK1 induces cisplatin resistance and apoptosis in glioblastoma cells [21,22]. It also decreases the phosphorylation levels of AKT/E1F4E, which occurs under certain hypoxic conditions [21]. Plexin B1 promotes SRPK1 activity through the PI3K/AKT signaling pathway, thereby increasing cell growth, angiogenesis, and motility in vitro and in vivo [21], promoting the malignant progression of tumors. The expression of SRPK1 is also closely associated with the response of various tumors to platinum-based chemotherapeutic drugs. Novel anti-tumor drugs targeting SRPK1 may be developed in the future [21,22]. However, the precise and effective molecular mechanisms underlying SRPK1 regulation of glioblastoma cell proliferation, invasion, and migration remain unclear, and the signaling pathways associated with SRPK1 have not been thoroughly explored. In-depth research on the mechanisms of glioblastoma occurrence and development, along with the discovery of new molecular mechanisms, is crucial for improving treatment outcomes and prognosis for glioblastoma patients. In our study, we found that after knocking down SRPK1, the Wnt/β-catenin and JAK-2/STAT-3 signaling pathways showed significant changes. This suggests a close association between SRPK1 and the Wnt/β-catenin and JAK-2/STAT-3 signaling pathways. In subsequent experiments, we confirmed that SRPK1 can participate in the malignant progression of glioblastoma by regulating the Wnt/β-catenin and JAK-2/STAT-3 pathways. This is a novel finding not previously reported in the literature. We believe that understanding the signaling pathways through which SRPK1 regulates the occurrence and development of glioblastoma is of great importance and provides a theoretical basis for the development of targeted therapies against SRPK1. Additionally, in this study, we established a xenograft mouse model and confirmed the role of SRPK1 in the tumor growth of xenografts in mice. Our experiments were systematic and revealed the role of SRPK1 in glioblastoma, elucidating its specific regulatory pathways and validating them using animal models.

The Wnt/β-catenin pathway is an important signaling pathway that plays a crucial role in embryonic development, cell proliferation, cell fate determination, and the physiological functions of adult cells [35]. The relationship between the Wnt/β-catenin pathway and tumors is very close, as it plays an important role in the occurrence, development, and metastasis of tumors. Aberrant activation of the Wnt/β-catenin pathway is closely associated with the occurrence of various tumors [36]. For example, abnormal activation of this pathway is commonly observed in liver cancer, colorectal cancer, and breast cancer, leading to the abnormal expression of tumor-related genes and increased cell proliferation. The JAK-2/STAT-3 signaling pathway is an important cellular signaling pathway [37,38], playing a crucial role in physiological processes such as cell proliferation, differentiation, apoptosis, and immune response. Aberrant activation of the JAK-2/STAT-3 signaling pathway is closely associated with the occurrence and development of various tumors. For example, abnormal activation of this pathway is commonly observed in breast cancer, lung cancer, gastric cancer, lymphoma, and other tumors. However, it has not been reported in gliomas.

In this study, we noticed that the results of transcriptome sequencing after knocking out SRPK1 indicated a significant decrease in the activity of the Wnt/β-catenin and JAK-2/STAT-3 signaling pathways, suggesting that SRPK1 may directly regulate Wnt/β-catenin and JAK-2/STAT-3 to promote the occurrence of gliomas. Silencing SRPK1 significantly reduced the activity of the Wnt/β-catenin and JAK-2/STAT-3 signaling pathways, while overexpression of SRPK1 significantly enhanced the activity of these pathways. Additionally, overexpression of SRPK1 rescued the decreased cell proliferation and migration and invasion abilities caused by silencing β-catenin or JAK-2. Subcutaneous tumor transplantation experiments in nude mice also showed that silencing SRPK1 significantly reduced the tumorigenic ability. These results all indicate that SRPK1 plays an important role in the occurrence of gliomas and that high expression of SRPK1 enhances the malignant phenotype of gliomas. SRPK1 promotes the proliferation, migration, and invasion of gliomas by activating the Wnt/β-catenin and JAK-2/STAT-3 signaling pathways.

This study provides a novel molecular marker, SRPK1, for the diagnosis and treatment of gliomas. The detection of SRPK1 can guide the selection of treatment plans, and personalized treatment strategies can be adjusted based on the levels of the patient’s biomarkers to improve treatment effectiveness. It can also be used to monitor disease progression and treatment effectiveness. Regular monitoring of changes in biomarkers can assess the effectiveness of treatment and adjust treatment plans in a timely manner. It also provides a potential therapeutic target for the treatment of glioblastomas.

However, this study also has some limitations. For example, whether SRPK1 has interacting partner molecules that jointly guide the occurrence and development of gliomas, whether knocking out SRPK1 will induce cell autophagy and ferroptosis, and whether these forms of cell death are related to the Wnt/β-catenin and JAK-2/STAT-3 signaling pathways, are all worth further investigation. Many viruses have been reported to be closely associated with tumor development, such as the Epstein–Barr virus (EBV), also known as human herpesvirus 4. EBV can transform naïve B cells into immortalized cells by regulating the cell cycle, cell proliferation, and apoptosis in vitro [39]. EBV infection is implicated in the development of various tumors. Whether the virus induces glioblastoma and whether SRPK1 is synergistically associated with virus-induced tumors are still subjects that require further investigation.

In conclusion, the expression of SRPK1 is positively correlated with the grade of gliomas and negatively correlated with patient prognosis, serving as an independent prognostic indicator. SRPK1 regulates the proliferation, invasion, and migration of glioma cells through the Wnt/β-catenin/JAK-2/STAT-3 pathway. As a novel biomarker for gliomas, SRPK1 provides a potential target for the treatment of gliomas.

## Figures and Tables

**Table 1:** Univariate and multivariate cox regression analysis of survival time in patients with WHO grade IV glioblastoma.

Variables	Uni HR			Multi HR		
	HR	95% CI	*p*	HR	95% CI	*p*
Gender (male, female)	0.93	0.75–1.35	0.765	-	-	-
Age (<60, ≥60)	0.97	0.85–1.25	0.151	-	-	-
KPS (<70, ≥70)	0.73	0.35–0.80	0.001	0.62	0.40–1.12	0.055
Radio-chemotherapy (yes, no)	0.27	0.18–0.35	<0.001	0.33	0.22–0.39	<0.001
SRPK1 (high, low)	1.86	1.25–2.50	<0.001	1.55	1.05–2.54	0.005

## Data Availability

The datasets used and analyzed during the current study are available from the corresponding author on reasonable request.

## Supplementary Materials

The following supporting information can be downloaded at: https://www.mdpi.com/article/10.3390/biomedicines12020378/s1, Table S1: Antibody information; Table S2: Baseline information of selected gliomas; Table S3: Primer Information; Figure S1: Immunofluorescence detection of morphological changes of SYNPO and PXN after knockdown of SRPK1 in U118MG and LN229 cells; Figure S2: Apoptotic vesicles after knockdown of SRPK1 in U118MG and LN229 cells; Figure S3: Scatterplot of genes from transcriptome sequencing after knockdown of SRPK1 in LN229 cells; Figure S4: Signaling pathway enrichment maps from transcriptome sequencing after knockdown of SRPK1 in LN229 cells; Figure S5: The proliferation of U343MG cells after SRPK1 knockout was detected by CCK-8; Figure S6: Detection of invasion and migration ability of knockout JAK-2 cells in U343 cells after knockout of JAK-2 or overexpression of SRPK1; Figure S7: CDH1 and VIM were detected by immunofluorescence in U343 cells after SRPK1 knockdown.
