# Supplementary material for: SRPK1 Promotes Glioma Proliferation, Migration, and Invasion through Activation of Wnt/β-Catenin and JAK-2/STAT-3 Signaling Pathways
Source: Biomedicines. 2024 Feb 6;12(2):378. doi: 10.3390/biomedicines12020378 (PMC10886746; doi:10.3390/biomedicines12020378)
Supplement: Supplementary file 1 [file biomedicines-12-00378-s001.zip › biomedicines-2776139-supplementary.pdf]

Supplementary Table S1. Antibody information

Antibody	Species	Vendor (City, State)	Dilution		
			WB	IF	IHC
GAPDH	Mouse	Proteintech (Wuhan, China)	1:5000	ND	ND
E-cadherin	Rabbit	Cell Signaling (Danvers, MA)	1:1000	1:200	ND
N-cadherin	Rabbit	Cell Signaling (Danvers, MA)	1:1000	1:200	1:200
Vimentin	Mouse	Cell Signaling (Danvers, MA)	1:1000	1:200	ND
BCL2	Rabbit	Cell Signaling (Danvers, MA)	1:1000	ND	ND
BAX	Mouse	Proteintech (Wuhan, China)	1:1000	ND	ND
CASP3	Rabbit	Sigma (Shanghai, CH)	1:1000	ND	ND
α-tubulin	Mouse	Abcam (San Francisco, CA)	ND	1:100	ND
γ-tubulin	Rabbit	Sigma (Shanghai, China)	1:1000	1:100	1:200
SRPK1	Rabbit	Cell Signaling (Danvers, MA)	1:1000	1:200	ND
p-JAK2	Rabbit	Cell Signaling (Danvers, MA)	1:1000	1:200	1:200
β-catenin	Rabbit	Abcam (San Francisco, CA)	1:1000	1:200	1:200
p-STAT3	Rabbit	Abcam (San Francisco, CA)	1:1000	1:200	1:200
JAK2	Rabbit	Abcam (San Francisco, CA)	1:1000	ND	ND

ND = Not detected; WB = Western blot; IF = immunofluorescence; IHC= Immunohistochemistry

Supplementary Table S2

Baseline information of selected gliomas.

Variable	No.(%)
Gender	
Male	122 (58.3%)
Female	88 (41.7%)
Age (years)	
≤60	159 (75.8%)
>60	51 (24.2%)
KPS	
<70	41 (19.4%)
>=70	119 (56.7%)
NA	50 (23.9%)
Grade	
2	26 (12.6%)
3	32 (15.0%)
4	152 (72.4%)

Radiaton/chemotherapy	
Yes	150 (71.5%)
No	60 (28.5%)

Supplementary Table S3

Primer name	Primer sequence
BCL2-F	ACTGGCTCTGTCTGAGTAAG
BCL2-R	CCTGATGCTCTGGGTAAC
GAPDH-F	GGGAAGGTGAAGGTCGGAGT
GAPDH-R	GGGGTCATTGATGGCAACA
BAX-F	GCTTCAGGGTTTCATCCAGG
BAX-R	GAGACACTCGCTCAGCTTCTTG
Caspase3-F	TTCATTATTCAGGCCTGCCGAGG
Caspase3-R	TTCTGACAGGCCATGTCATCCTCA

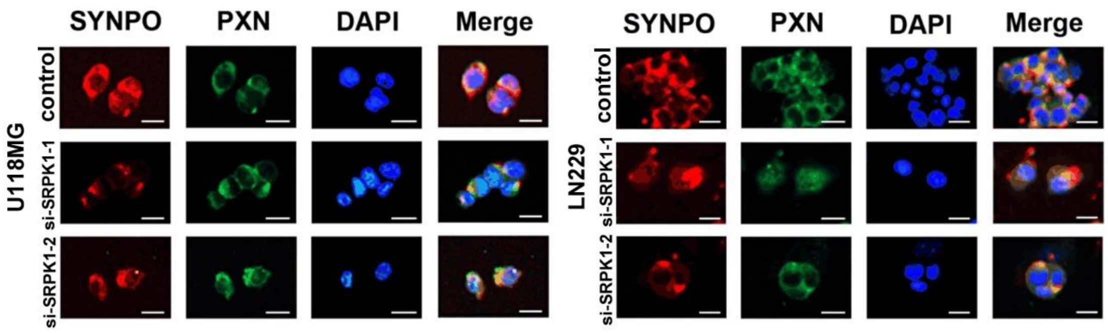

Figure S1. Immunofluorescence detection of morphological changes of SYNPO and PXN after knockdown of SRPK1 in U118MG and LN229 cells

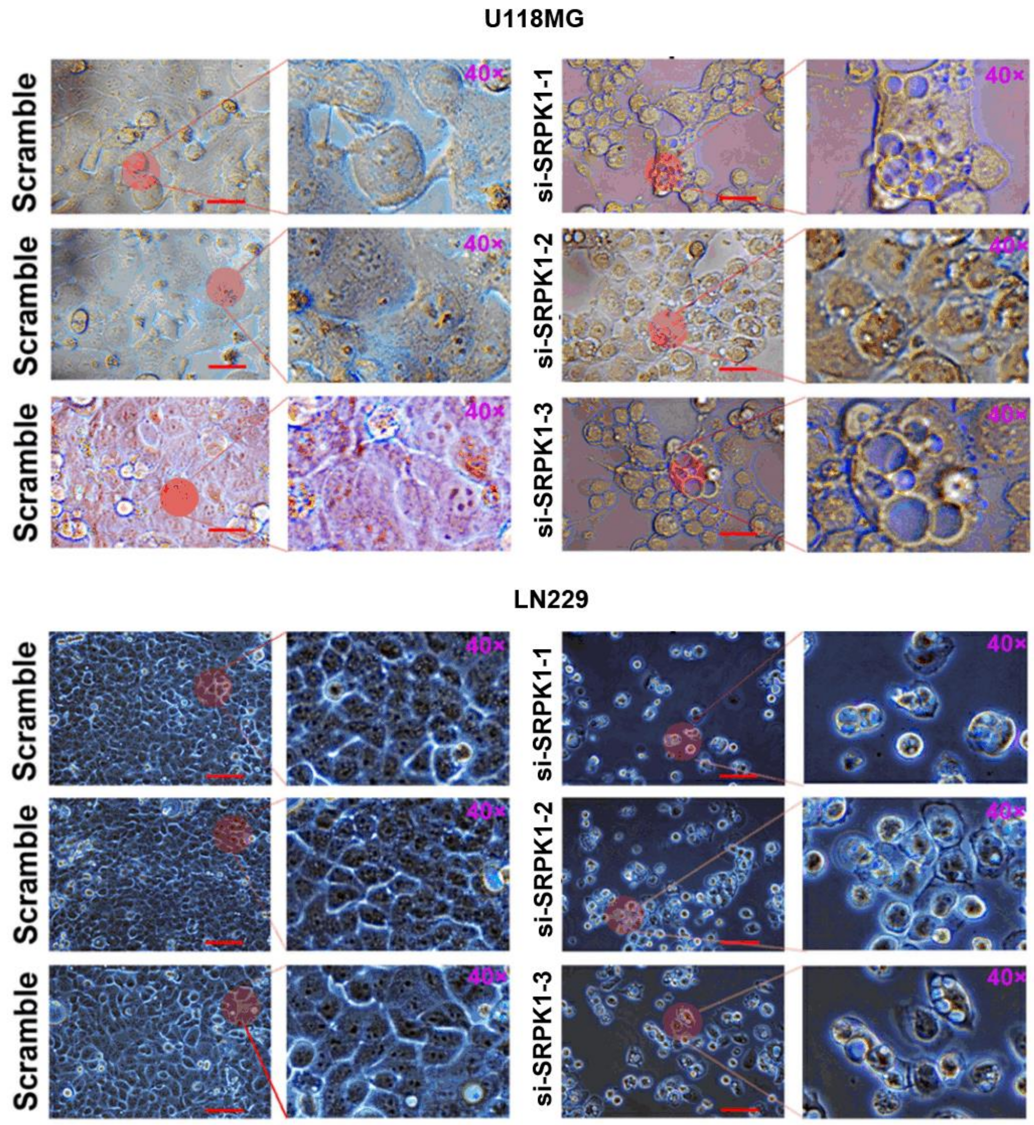

Figure S2. Apoptotic vesicles after knockdown of SRPK1 in U118MG and LN229 cells

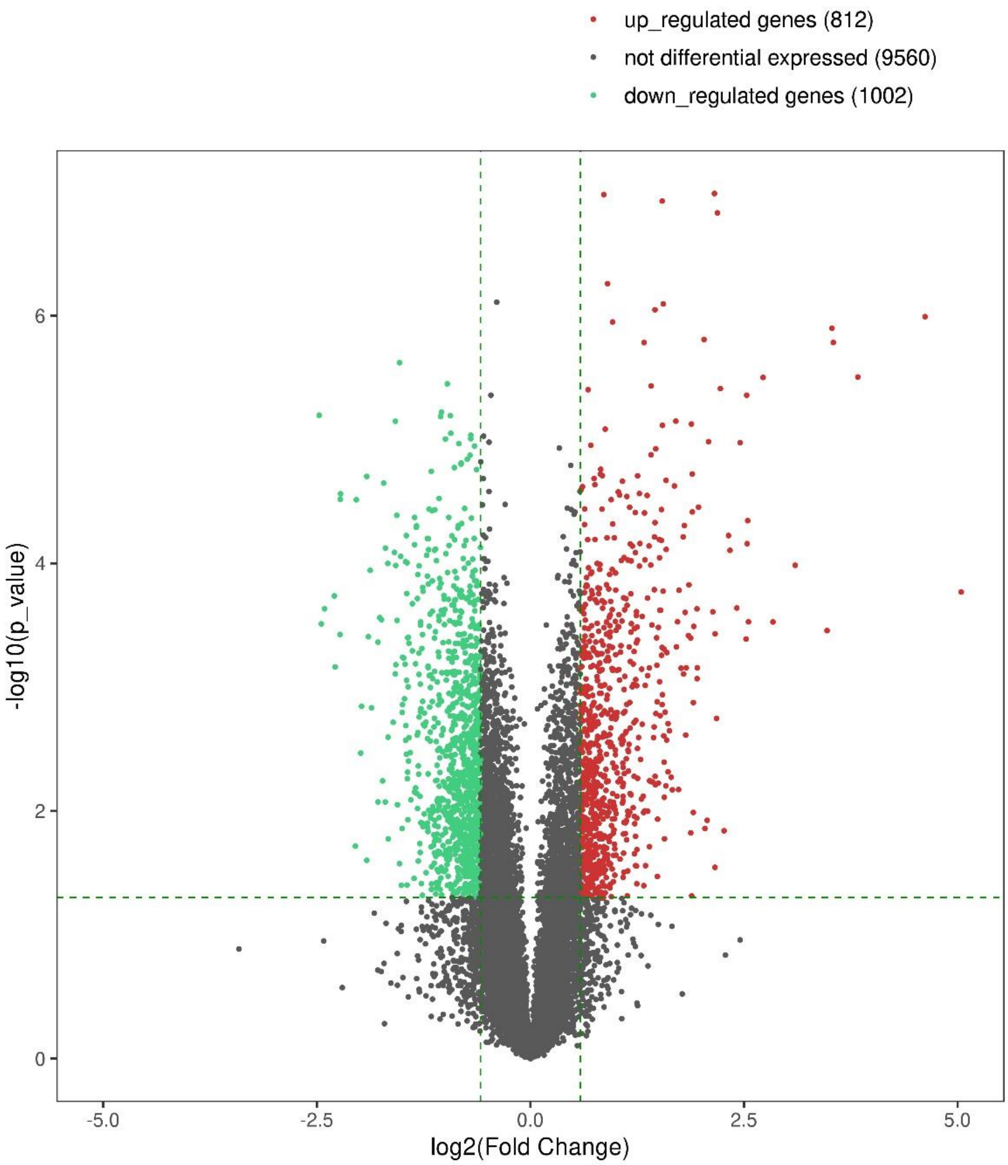

Figure S3. Scatterplot of genes from transcriptome sequencing after knockdown of SRPK1 in LN229 cells

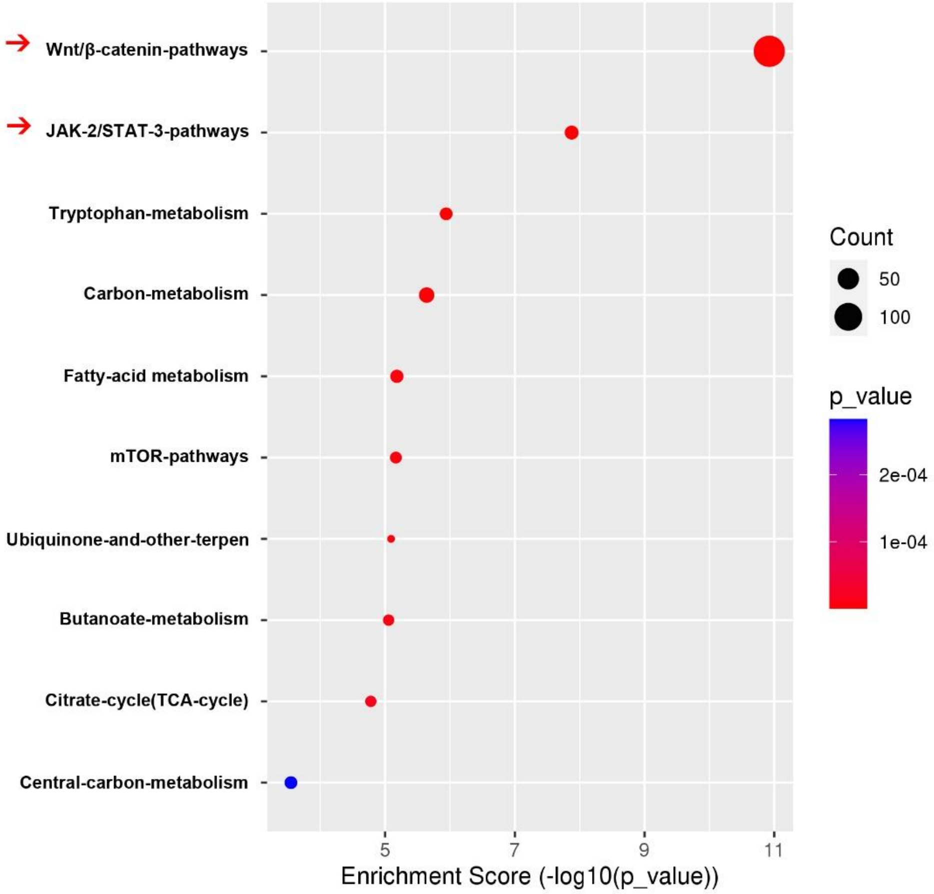

Figure S4. Signaling pathway enrichment maps from transcriptome sequencing after knockdown of SRPK1 in LN229 cells

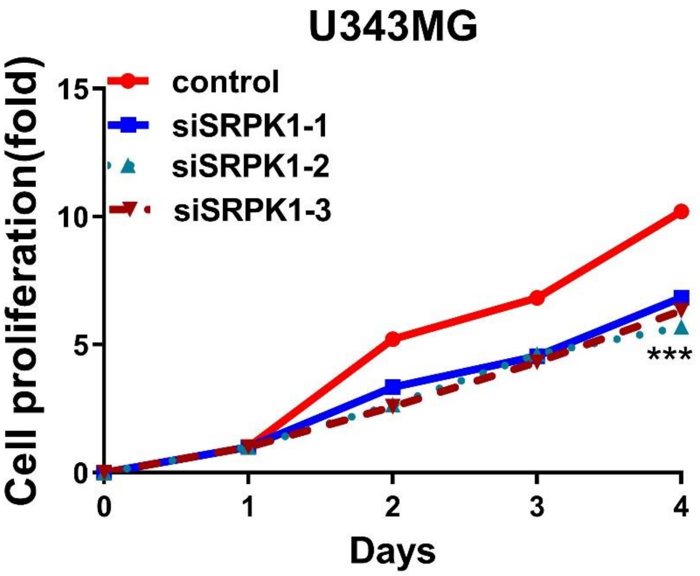

Figure S5. The proliferation of U343MG cells after SRPK1 knockout was detected by CCK-8

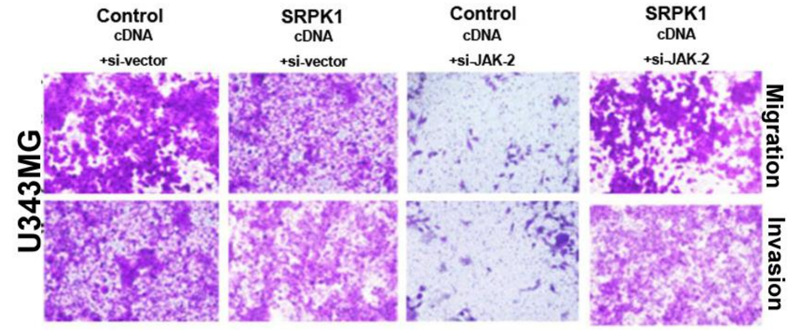

Figure S6. Detection of invasion and migration ability of knockout JAK-2 cells in U343 cells after knockout of JAK-2 or overexpression of SRPK1

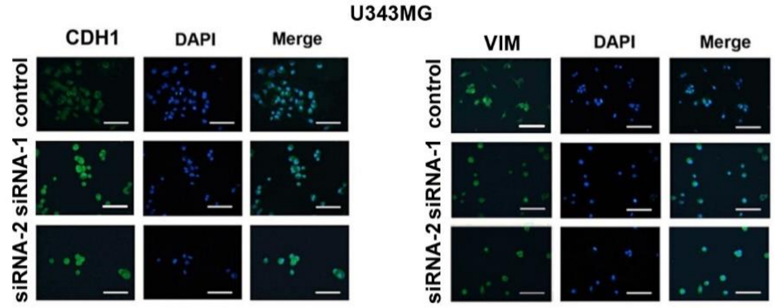

Figure S7. CDH1 and VIM were detected by immunofluorescence in U343 cells after SRPK1 knockdown
